# Inhibition of triosephosphate isomerase by phosphoenolpyruvate in the feedback-regulation of glycolysis

**DOI:** 10.1098/rsob.130232

**Published:** 2014-03-05

**Authors:** Nana-Maria Grüning, Dijun Du, Markus A. Keller, Ben F. Luisi, Markus Ralser

**Affiliations:** 1 Department of Biochemistry, University of Cambridge, 80 Tennis Court Road, Cambridge CB2 1GA, UK; 2 Cambridge Systems Biology Centre, University of Cambridge, 80 Tennis Court Road, Cambridge CB2 1GA, UK; 3 Division of Physiology and Metabolism, MRC National Institute for Medical Research, The Ridgeway, Mill Hill, London NW7 1AA, UK

**Keywords:** glycolysis, triosephosphate isomerase, phosphoenolpyruvate, pyruvate kinase, feedback loop

## Abstract

The inhibition of triosephosphate isomerase (TPI) in glycolysis by the pyruvate kinase (PK) substrate phosphoenolpyruvate (PEP) results in a newly discovered feedback loop that counters oxidative stress in cancer and actively respiring cells. The mechanism underlying this inhibition is illuminated by the co-crystal structure of TPI with bound PEP at 1.6 Å resolution, and by mutational studies guided by the crystallographic results. PEP is bound to the catalytic pocket of TPI and occludes substrate, which accounts for the observation that PEP competitively inhibits the interconversion of glyceraldehyde-3-phosphate and dihydroxyacetone phosphate. Replacing an isoleucine residue located in the catalytic pocket of TPI with valine or threonine altered binding of substrates and PEP, reducing TPI activity *in vitro* and *in vivo*. Confirming a TPI-mediated activation of the pentose phosphate pathway (PPP), transgenic yeast cells expressing these TPI mutations accumulate greater levels of PPP intermediates and have altered stress resistance, mimicking the activation of the PK–TPI feedback loop. These results support a model in which glycolytic regulation requires direct catalytic inhibition of TPI by the pyruvate kinase substrate PEP, mediating a protective metabolic self-reconfiguration of central metabolism under conditions of oxidative stress.

## 2. Introduction

With the challenge of surviving in a constantly changing environment, cells have evolved mechanisms to flexibly regulate metabolism [1,2]. An important and dynamically regulated metabolic pathway is glycolysis, an ancient chemical route of carbohydrate utilization that produces ATP, NADH and intermediate metabolites for the synthesis of nucleotides, fatty acids and amino acids. Glycolysis is mainly regulated through feedback and feed-forward cycles involving its intermediate metabolites. These cycles sustain intermediates while preventing their accumulation to toxic levels and are responsible for the oscillating behaviour of glycolytic reactions [3–6]. Moreover, this enzymatic regulation is important for maintaining the balance of metabolism during changes in cell growth or environment [1,2]. As an example, the increased need for the redox cofactor NADPH during oxidative stress caused upon hydroperoxide exposure is met by diverting glycolytic flux into the pentose phosphate pathway (PPP). This transition is rapidly inducible by metabolic inhibition of glycolysis, changes in the activity of glucose 6-phosphate dehydrogenase (the first enzyme of the oxidative PPP), followed by transcriptional control during mid- to long-term adaptation to oxidative conditions [7–10].

A similar mechanism acts to prevent an accumulation of oxidizing metabolites in cancer cells or cells that respire at high rates. These frequently possess a higher activity of the PPP to balance the greater demand for NADPH by the anti-oxidant machinery and to compensate for the increased production of reactive oxygen species [11,12]. Current findings have highlighted the importance of the terminal glycolytic enzyme pyruvate kinase (PK) to achieve the regulation of glycolysis and the PPP. Low activity of PK has been found in cancer and rapidly proliferating cells, and in yeast cells with high respiration activity [13,14]. More recently, it has been proposed that cancer cells profit from the loss of the PKM2 gene during tumour formation [15]. Reduced PK activity caused accumulation of its substrate, phosphoenolpyruvate (PEP), which correlates with an increased activity of the PPP [14,16], and increased oxidant tolerances of both mammalian and yeast cells [11,14]. It has been observed that PEP is an inhibitor of another metabolic redox regulator, triosephosphate isomerase (TPI or TIM, EC 5.3.1.1) [17,18]. In its glycolytic role, TPI is regarded as a near-perfect catalyst because its catalytic speed *in vitro* only depends on the rate of diffusion of its substrates [19]. *In vivo*, TPI interconverts dihydroxyacetone phosphate (DHAP) and glyceraldehyde-3-phosphate (G3P) to prevent an accumulation of DHAP [19,20]. Reduced activity of TPI in yeast and *Caenorhabditis elegans* leads to a partial inhibition of glycolysis but is beneficial during oxidative stress, as it increases the concentration of PPP metabolites and stress tolerance in both species [7,21]. We have shown previously that the increased oxidative stress resistance of PK mutants is attributable to *TPI* as well. In yeast cells expressing mutant TPI with lowered activity, PK failed to increase stress resistance, while a deletion of the first enzyme of the oxidative PPP, glucose 6-phosphate dehydrogenase (*G6PDH, ZWF1*), leads to protein and mitochondrial oxidative damage in a PK-dependent manner [14].

To understand how PEP affects TPI activity, we generated a co-crystal structure of the enzyme in complex with PEP at 1.6 Å resolution. We find that PEP directly interacts with TPI by binding into the catalytic pocket of the enzyme and outcompetes the substrates from their binding position. Moreover, the structural data reveal that PEP interacts with the conserved Ile170, a residue which when mutated is associated with TPI deficiency in humans [22], and in yeast affects response to oxidative stress [7,23] and PK function [14]. We use this mutant and others inferred from the crystallographic structure to define the kinetics and stability properties of TPI upon PEP binding. We demonstrate that the *in vivo* consequence of competitive TPI inhibition is the activation of the PPP and altered stress resistance.

## 3. Results and discussion

### 3.1. Structure of the triosephosphate isomerase–phosphoenolpyruvate complex

**Figure 1.**
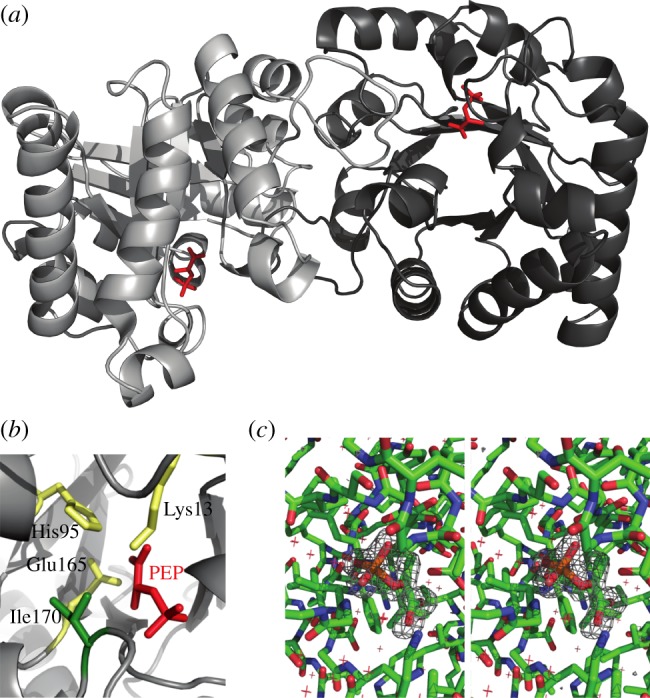
Co-crystal structure of TPI with bound PEP. (*a*) Schematic of the TPI–PEP crystallographic structure. PEP locates in the active centre of both subunits in the asymmetric TPI dimer. (*b*) The catalytic pocket of TPI bound to PEP. Catalytic residues are highlighted in yellow, PEP in red, isoleucine 170 in green. (*c*) Stereoscopic illustration of the PEP binding site environment including a difference map in which PEP has been removed from the model and was refined against the experimental data for five cycles. The map has been contoured at 4 s.d. and reveals positive density for the missing ligand.

**Figure 2.**
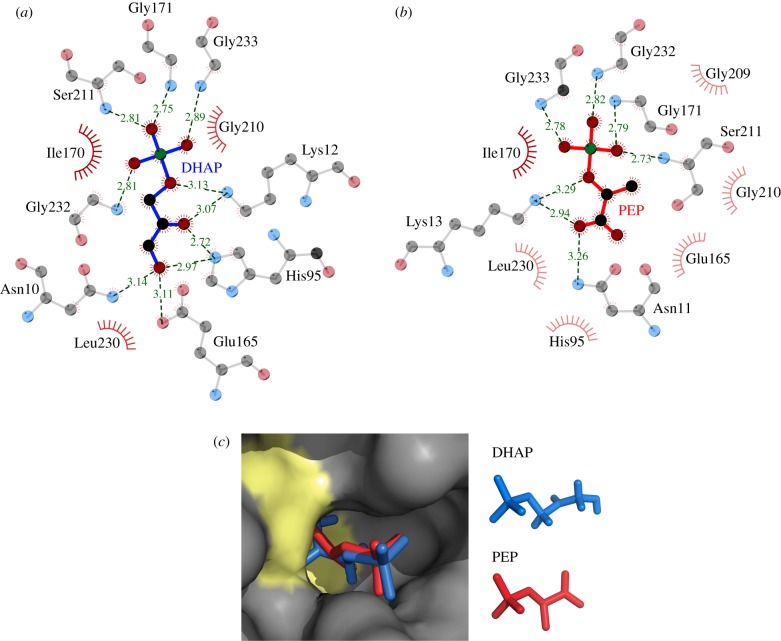
The TPI inhibitor PEP and the TPI substrate DHAP have similar interaction sites. (*a*) Contact distances between TPI and its substrate DHAP, and (*b*) the interactions of TPI and PEP in the active site. PEP and DHAP are in contact with similar principal residues. Distances are given in Å. Green balls, phosphate; grey balls, carbon; red balls, oxygen. The red circles indicate residues in close proximity to the ligand. Illustrations were prepared using LigPlot. (*c*) PEP and DHAP bind similarly to the TPI active site. Rabbit TPI bound to PEP, overlaid with the location of the TPI substrate DHAP as determined by Jogl *et al.* [28] as surface representation. Yellow areas highlight catalytically active residues; PEP: red; DHAP: blue.

TPI is a ubiquitous enzyme with homologues found throughout all kingdoms of life [20,24] and that in human populations possesses only a minimum of sequence divergence [25]. To study the TPI–PEP interaction, we co-crystallized PEP and rabbit TPI, which differs from human TPI in four non-conserved residues only (electronic supplementary material, figure S1). The structure was solved by molecular replacement and refined at 1.55 Å resolution (table 1). The asymmetric unit contains a homodimer of TPI (figure 1*a*). Each protomer contains eight α-helices on the outside and eight parallel β-strands on the inside, forming a typical TIM-barrel [26]. Comparison of TPI-PEP with a previously reported structure of rabbit muscle *apo* TPI [27] shows that the active site loops are in the closed conformation in both subunits. The electron density map gave a clearly defined and unambiguous shape for PEP bound to the active sites of both subunits (figure 1*a*,*c*). Active site residues engage PEP and make similar interactions to those observed for the TPI substrate DHAP (figures 1*b* and 2*a,b*; a stereoscopic illustration is given in figure 1*c*) [28]. For substrate conversion, TPI employs a catalytic triad consisting of the residues Lys13, His95 and Glu165 [27], whereas PEP is in contact with the catalytically active residue Lys13 and the active site residues Gly232, Gly233, Gly171, Ser211 and Asn11 (figure 2). The positioning of PEP thus indicates that it binds into the catalytic pocket of TPI and competes with the substrates for binding with catalytic residues (figure 2*c*).

**Table 1.** Crystallographic data collection and refinement statistics. The PDB deposition code for model and structure factors of TPI–PEP is 4OWG.

data collection	
wavelength (Å)	0.9795
resolution (Å)	43.1–1.55
(high-resolution shell)	1.63–1.55
*R*_merge_	0.086 (0.314)
unique reflections	59 113
completeness	93.4
multiplicity	2.7
*I*/*σ*(*I*)	8.0
unit cell (*a*, *b*, *c* (Å); *α*,*β*,*γ* (deg))	*a* = 36.85, *b* = 72.07, *c* = 161.20, *α* = *β* = *γ* = 90
space group	P2_1_2_1_2_1_
refinement	
*R* (working set)	0.1665
*R*_free_ (test set)	0.2032
*RMS deviations*	
bond lengths (Å)	0.0195
bond angles (Å)	1.9061
*Ramachandran statistics*	
% of residues in allowed regions	465 (96.27%)
% of residues in generously allowed	14 (2.90%)
% of residues in not allowed	4 (0.83%)
model	
amino acids TPI	246 of 250
water molecules	432

### 3.2. Structure–function analysis of the triosephosphate isomerase–phosphoenolpyruvate interaction

We observed that PEP is in direct contact with a conserved isoleucine at position 170. A human TPI allele mutant for this residue (Ile170Val) has been found in a rare variant in the human genetic disorder TPI deficiency. This mutation translates into a mutant TPI with reduced catalytic activity [21,22]. TPI deficiency manifests as recessive autosomal multi-system disorder, which is caused by structural defects in the TPI enzyme [29]. Based on the crystallographic information, we predicted two further residue exchanges to affect PEP binding and generated two constructs encoding for TPI_Lys13Arg_ as well as TPI_Ile170Thr_. Lys13 is known to be required for the catalytic mechanism [26] and exchanging it to arginine rendered the enzyme not only catalytically inactive but also largely unstable (electronic supplementary material, figure S3; figures 4 and 5). Thus, our analyses shown below focused mostly on the TPI_Ile170Val_ and TPI_Ilel70Thr_ proteins that retained stability and residual catalytic activity.

### 3.3. TPI_Ile170Val_ and TPI_Ile170Thr_ exhibit altered phosphoenolpyruvate and glyceraldehyde-3-phosphate binding

**Figure 3.**
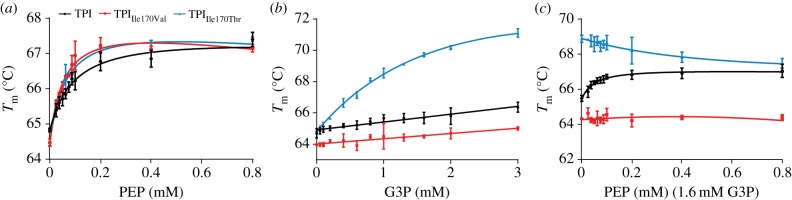
PEP competes with G3P for binding to human TPI. (*a*) Thermal stability of human TPI and active-site mutants TPI_Ile170Val_ and TPI_Ile170Thr_ in the presence of increasing PEP concentrations. PEP stabilized the three-enzyme species indicative for binding; TPI_Ile170Val_ and TPI_Ile170Thr_ were stabilized to an increased extent. (*b*) Thermal stability of human TPI mutants to increasing G3P concentrations; increased thermal stability of TPI_Ile170Thr_ indicated augmented affinity for G3P. (*c*) PEP dose–response curve in the presence of G3P. PEP binding was competitive against G3P in human TPI and TPI_Ile170Thr_, but did not influence the thermal stability of TPI_Ile170Val_.

We expressed 6*x*-histidine tagged wild-type human TPI, TPI_Ile170Val_, TPI_Ile170Thr_ and TPI_Lys13Arg_ in *Escherichia coli* and purified the enzymes using metal affinity chromatography. Far-UV circular dichroism (CD) spectroscopy of the purified recombinant enzymes showed a similar composition of secondary structures, indicating that the mutations did not prevent folding (electronic supplementary material, figure S2). To determine the impact of the mutations on the interactions of TPI with PEP and G3P, we conducted thermal melt assays using the fluorescent probe SYPRO Orange [30]. In the presence of incremental PEP concentrations, TPI and its mutants exhibited thermo stabilization, indicating that the proteins bound the metabolite (figure 3*a*). Interestingly, TPI_Ile170Val_ and TPI_Ile170Thr_ responded more strongly to the presence of PEP (TPI_Ile170Val_ Δ_Tm_ = 2.64°C, TPI_Ile170Thr_ Δ_Tm_ = 2.95°C) in comparison with a Δ_Tm_ = 2.57°C for human wild-type TPI, indicating that the mutations increased the binding affinity to PEP (figure 3*a*).

Next, we assessed structural stability in the presence of the TPI substrate, G3P. This substrate is expected to be constantly metabolized to DHAP (and back) [31,32], and adding up to 3 mM G3P to wild-type TPI caused a slight increase in the enzyme's thermal stability (figure 3*b*). The effects of G3P addition to TPI_Ile170Val_ were comparable with that of the wild-type. A much stronger response was however observed for TPI_Ile170Thr_. This mutant substantially gained stability in the presence of G3P (figure 3*b*; Δ_Tm_ = 6.21°C at 3 mM G3P, wild-type TPI Δ_Tm_ = 1.68°C), indicating that the binding affinity to this substrate was increased. We speculate that the increased substrate affinity is facilitated by a hydrogen bond between the substrate and the threonine side chain.

Finally, we tested whether protein stability is affected by PEP in the presence of G3P. In the wild-type form, PEP was competitive with G3P for binding the enzyme, as expressed by an increase in thermal stability even at PEP levels lower than 0.25 mM (figure 3*c*). Conversely, the increased thermostability mediated by G3P specifically to the TPI_Ile170Thr_ enzyme (figure 3*b*) was partially lost upon adding PEP (figure 3*c*), confirming competitive binding in this mutant as well. By contrast, TPI_Ile170Val_ was resistant to increased PEP levels (figure 3*c*), indicating that this metabolite was no longer competitive for binding. In summary, thermal shift assays confirmed binding of PEP to TPI. The different behaviour of the TPI_Ile170Val_ and TPI_Ile170Thr_ mutants in this process supports the crystallographic identification of the binding site to be the catalytic pocket and indicates direct contact of PEP and G3P with this isoleucine residue.

### 3.4. Inhibitory effects of phosphoenolpyruvate on triosephosphate isomerase catalysis

**Figure 4.**
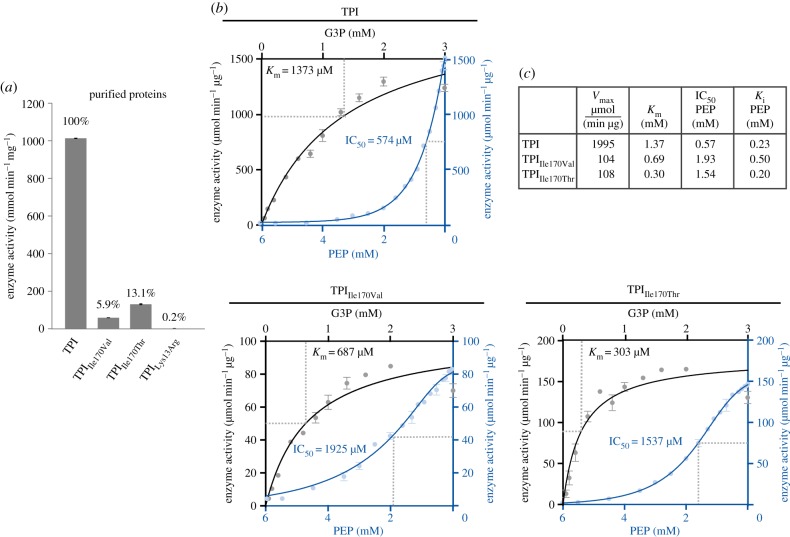
PEP inhibits the catalytic activity of TPI. (*a*) TPI_Ile170Val_ and TPI_Ile170Thr_ have reduced catalytic activity, TPI_Lys13Arg_ is inactive. Enzyme activity expressed as substrate conversion rate in micromoles per minute and microgram protein. (*b*) Enzymatic properties of TPI, TPI_Ile170Val_, TPI_Ile170Thr_ and their inhibition by PEP. (*c*) Substrate titration curves of G3P (black curves, to be read from left to right) on TPI and its mutant enzymes, as well as inhibitor titration curves for PEP (blue curves, to be read from right to left). Substrate/inhibitor saturation was used to calculate *V*_max_, *K*_m_ (G3P titrations), and IC_50_ and *K*_i_ values (PEP titrations) (inset table).

Next, we performed enzyme-coupled assays to determine changes in the catalytic activity of TPI in the mutants as well as in the presence of PEP. The three mutations affecting residues located in the catalytic pocket, TPI_Ile170Val_, TPI_Ile170Thr_ and TPI_Lys13Arg_, all reduced the catalytic activity of TPI (figure 4*a*). The substantial residual activities of 5.9% for TPI_Ile170Val_ or 13.1% of TPI_Ile170Thr_ indicate that Ile170 is not essential for TPI's catalytic function. By contrast, TPI_Lys13Arg_ exhibited only catalytic activity around the detection limit of the assay (approx. 0.2% compared with wild-type level), confirming that Lys13 is essential for catalysis, as reported earlier [33].

To quantify enzymatic activity for wild-type TPI, TPI_Ile170Val_ and TPI_Ile170Thr_, and to determine the rate of their inhibition by PEP (expressed as *K*_i_, IC_50_ values), we generated substrate saturation and PEP titration curves for these enzyme species. The mutant enzymes exhibited lower substrate conversion rates and saturated at lower concentrations of G3P (*K*_m_ for wild-type TPI: 1373 µM, TPI_Ile170Val_: 687 µM and TPI_Ile170Thr_: 303 µM). This indicates that despite its lower activity, the TPI_Ile170Thr_ mutant had higher affinity to the TPI substrate (figure 4*b*, black curves, from left to right). Next, we titrated PEP to the reaction operating at maximal activity. In all cases, a strong and concentration-dependent inhibition of the enzyme activity was observed. In the case of human wild-type TPI, 50% of enzyme activity was lost in the presence of 570 µM PEP (IC_50_), corresponding to a *K*_i_ of 230 µM (figure 4*b*, blue curves, to be read from right to left). Compared with pharmacological inhibitors, PEP is thus a relatively low-affinity inhibitor for TPI. However, this appears biologically meaningful, as PEP is constantly present at high cellular levels [14]. A high affinity for PEP would thus render TPI constantly inactive.

Finally, we observed that the introduced TPI mutations influenced the PEP sensitivity of TPI. The mutant species exhibited strongly increased IC_50_ values (TPI_Ile70Val_ = 1925 µM, TPI_Ile170Thr_ = 1537 µM). This finding supports the crystallographic result that isoleucine 170 interacts with PEP, rendering the mutant enzymes in relative terms more PEP-resistant.

### 3.5. *In vivo* complementation of triosephosphate isomerase, TPI_Ile170Val_ and TPI_Ile170Thr_

**Figure 5.**
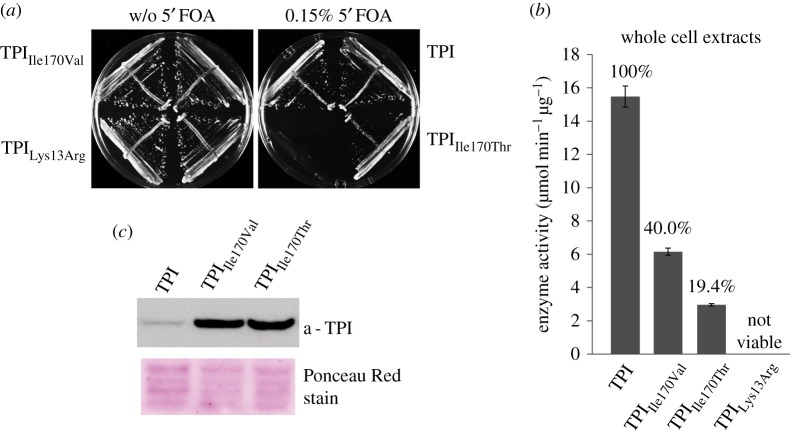
Human TPI_Ile170Val_ and TPI_Ile170Thr_ complement for yeast TPI and are catalytically active. (*a*) TPI, TPI_Ile170Val_ and TPI_Ile170Thr_, but not TPI_Lys13Arg_, complement for yeast *TPI1*. In a plasmid shuffle experiment, Δ*tpi1* cells carrying a counterselectable TPI-encoding plasmid were transformed with a centromeric plasmid (minichromosome) encoding the indicated TPI mutants. Transformed cells were then transferred to 5'FOA to induce loss of the counterselectable plasmid. Only cells containing a functional TPI copy on the minichromosome are viable on glucose media after counterselection. Human TPI, TPI_Ile170Val_ and TPI_Ile170Thr_ complemented for a loss of the TPI plasmid, but TPI_Lys13Arg_ did not. (*b*) TPI activity in yeast whole-cell extracts. Substrate conversion rates as normalized to total protein content. TPI_Ile170Val_ and TPI_Ile170Thr_ have lower activity than wild-type TPI. (*c*) Increased expression levels of TPI_Ile170Val_ and TPI_Ile170Thr_ in yeast as revealed by immunoblotting of whole-cell extracts using a TPI-specific antibody [35]. The amount loaded onto the SDS-PAGE gel was normalized to total protein, comparable loading was evaluated by Ponceau Red staining of the blotting membrane.

In the next step, we tested whether cells carrying the mutant enzymes were capable of maintaining metabolism. For this, we used a yeast strain (MR100 [21]) chromosomally deleted for yeast *TPI1*, a direct sequence orthologue of human TPI catalysing the same reaction, and which is kept viable by expressing TPI from a 5'FOA-counterselectable *URA*3 plasmid. We introduced human TPI, TPI_Ile170Val_, TPI_Ile170Thr_ and TPI_Lys13Arg_ into this strain, then selected on 5'FOA media for cells that had lost the TPI-*URA3* plasmid. Yeast strains expressing wild-type TPI, TPI_Ile170Val_ and TPI_Ile170Thr_ could be cultured in glucose-containing media, indicating that these enzymes compensated for the loss of yeast TPI, demonstrating catalytic activity *in vivo.* By contrast, yeast cells expressing TPI_Lys13Arg_ were not viable, confirming that TPI_Lys13Arg_ was not catalytically functional (figure 5*a*).

Next, TPI activity was measured in cell extracts of the transgenic strains. As the total TPI substrate conversion per microgram protein in the cell extract corresponded to 1.5% compared to the pure enzyme (15.5 µmol NADH min^−1^ µg protein^−1^), we estimate that TPI accounts for approximately 1.5% of total soluble protein, substantiating that TPI is one of the most abundant cytoplasmic proteins [34]. Interestingly, we noted that the total activity of mutant enzymes (TPI_Ile170Val_, TPI_Ile170Thr_) was, relative to wild-type, significantly lower in their purified version compared with what we measured in the cell extracts (figures 4*a* and 5*b*). An analysis of TPI expression levels by immunoblotting using a specific TPI antisera [35] however revealed that mutant TPI is much more strongly expressed compared with wild-type TPI (figure 5*c*). This indicates that cells compensated for a loss of specific TPI activity by the upregulation of the enzyme abundance.

### 3.6. Low triosephosphate isomerase activity mediates elevation in pentose phosphate pathway metabolite concentrations, oxidant resistance and heat sensitivity

We have shown previously that reduced activity of TPI causes a re-configuration of central metabolism, leading to increased flux of the PPP and increased stress resistance in yeast and *C. elegans* [7]. The feedback inhibition of TPI by PEP is therefore expected to have similar consequences. In bacteria, yeast and mammalian cells, PEP accumulation is caused by a diminution of PK activity [11,14,16]. Whereas low PK activity in yeast is correlated with high respiration rates and superoxide production [14], in human cells it is associated with rapid cell proliferation and cancer [13,36]. Affected by high ROS production, cancer cells upregulate the allosterically regulated PK isoform PKM2 [37], which is redox-sensitive and the PKM isoform with lower catalytic activity [11,13,36]. Moreover, recent results have demonstrated that cancer cells have higher survival chances when they lose this gene [15]. This situation causes a block of the early steps of glycolysis and increases the PPP activity resulting in augmented oxidant tolerance of both yeast and mammalian cells [11,14], indicating that the PK–TPI feedback loop is important for oxidative stress protection.

**Figure 6.**
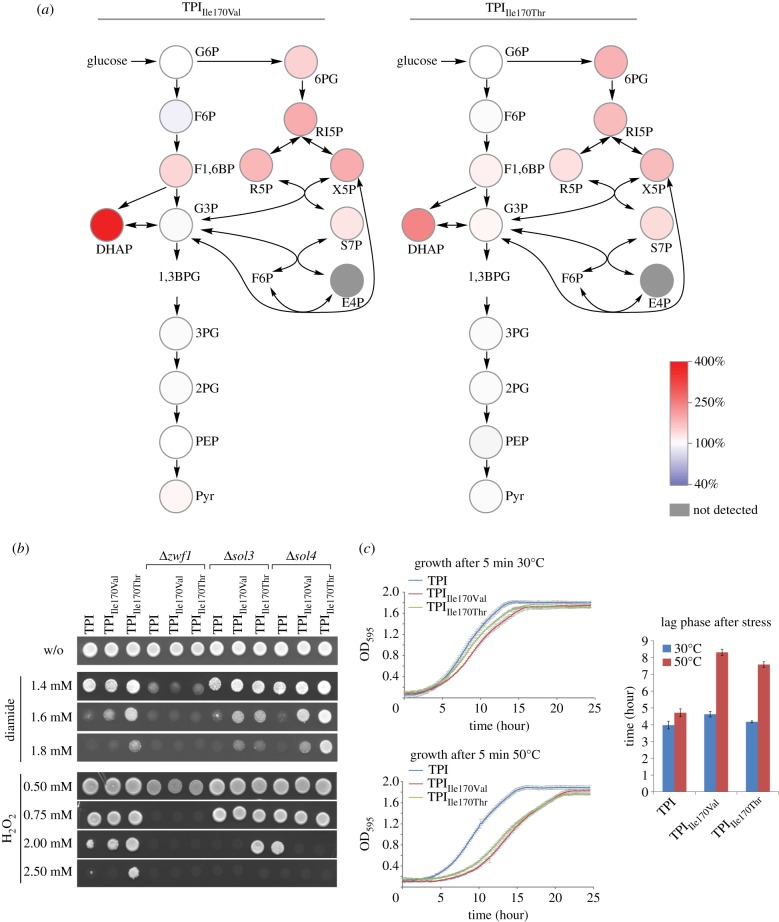
Low TPI activity increases PPP metabolite load and causes oxidant resistance and heat sensitivity. (*a*) Concentrations of glycolytic and PPP metabolites in the human TPI_Ile170Val_ and TPI_Ile170Thr_ mutants relative to yeast expressing human wild-type TPI. PPP and glycolytic metabolites were quantified by LC-MS/MS. PPP metabolites are increased in the TPI mutants. Absolute values are given in the electronic supplementary material, figure S4. (*b*) TPI_Ile170Val_ and TPI_Ile170Thr_ mediate increased tolerance to oxidizing agents. Overnight cultures of the indicated yeast strains were diluted to an OD_600_ = 3 and spotted onto SC^−His^ agar plates containing the oxidants. Glucose 6-phosphate dehydrogenase (Zwf1) encodes the enzyme for the first step in the non-reversible oxidative PPP shunt and produces NADPH. Its deletion abolishes the oxidant resistance phenotype of cells expressing TPI_Ile170Val_ or TPI_Ile170Thr_. Sol3 and Sol4 catalyse the second step of the PPP and their deletion reduced oxidant resistance on H_2_O_2_; a protective effect of TPI_Ile170Val_ was detected in Δ*sol3* yeast while causing H_2_O_2_ sensitivity in Δ*sol4* yeast. (*c*) TPI mutants are heat-sensitive. Overnight cultures were diluted to an OD_600_ = 0.2 and exposed, or not exposed, to 50°C for 5 min and growth was monitored for 25 h after heat exposure. The duration until growth was re-established (lag phase) was used as an inverse indicator for heat resistance. The lag phase was prolonged in the 50°C exposed TPI mutants compared with isogenic yeast cells expressing wild-type TPI.

As shown above, TPI substrates and PEP bind to the same structural site and have largely the same contact residues. As a consequence, mutations that affect PEP binding also affect the catalytic activity of TPI. This prevents the creation of an ideal *in vivo* model where TPI feedback inhibition by PEP would be disrupted while TPI catalytic activity is unaffected. However, the mutant proteins provide a means of studying the consequences of specifically lowered TPI activity that mimics the situation of feedback inhibition. We used the yeast strains expressing TPI_Ile170Val_ and TPI_Ile170Thr_ to determine glycolytic and PPP metabolite concentrations by liquid chromatography tandem mass spectrometry (LC-MS/MS), adapting our previous procedures [38,39]. In comparison with the isogenic strain expressing wild-type TPI, yeast cells expressing both the naturally occurring TPI_Ile170Val_ allele and the designed TPI_Ile170Thr_ protein displayed an increased concentration of PPP intermediates, indicating higher activity of this pathway confirming the previous results (figure 6*a*; electronic supplementary material, figure S4). Moreover, glycolytic intermediates upstream of TPI were affected, with the strongest measured increase in the concentration of the TPI substrate DHAP (figure 6*a*), reflecting the partial blockage of glycolysis.

Next, we tested for consequences of expressing the mutant TPI forms in regard to stress resistance. For this, the transgenic strains were rendered prototrophic by transformation with the pHLUM minichromosome [40]. Then, the cells were spotted on media containing the thiol-oxidizing compound diamide, as resistance to this compound has previously been shown to be dependent on PPP activation [7,41], and on media containing hydrogen peroxide, a naturally occurring oxidant. Resistance against both oxidants was increased in cells expressing TPI_Ile170Val_ and TPI_Ile170Thr_, with the effects being stronger for diamide (figure 6*b*). To address whether this phenotype was directly depending on the PPP, a similar set of experiments was then conducted in isogenic strains deleted for the gene encoding glucose 6-phosphate dehydrogenase (*ZWF1*), the first enzyme of the oxidative PPP that is a direct source of NADPH [42], and *SOL3* and *SOL4,* two paralogous genes which catalyse the next (non-NADP(H)-dependent) step of the pathway [43]. The deletion of *ZWF1* (on both oxidants) reduced yeast oxidant tolerances (figure 6*b*). The deletion of *SOL3* and *SOL4* caused weaker effects on H_2_O_2_ only (figure 6*b*). In combination with *ZWF1,* the protective effects of TPI_Ile170Val_ and TPI_Ile170Thr_ were lost, and this phenotype was affected in combination with *SOL3* and *SOL4* deletions as well (figure 6*b*). This indicates that the TPI-mediated oxidant protection is dependent on the oxidative PPP, and mainly on its first NADPH-producing enzyme, the glucose 6-phosphate dehydrogenase Zwf1p.

Yeast strains with low PK activity are resistant to oxidants [14] but sensitive to heat [44]. Therefore, we tested whether a similar behaviour was observed in the TPI mutant strains. Exponentially growing yeast strains were exposed to 50°C for five minutes or kept at 30°C and used to inoculate a fresh culture. The heat-induced growth delay, calculated using a model free spline fit [45], was used as a measure of yeast heat resistance. Yeast cells expressing human TPI well tolerated the heat treatment; however, yeast harbouring TPI_Ile170Val_ and TPI_Ile170Thr_ were heat-sensitive, resulting in a strong delay until growth resumed (figure 6*c*). Thus, low TPI activity, despite protecting against oxidants, causes heat sensitivity. In summary, similar to what has been observed in cells with low PK activity [14,44], expressing TPI_Ile170Val_ and TPI_Ile170Thr_ increased PPP metabolite concentrations and mediated oxidant resistance and heat sensitivity.

## 4. Conclusion

The central glycolytic enzyme TPI plays a crucial role in coordinating energy with redox metabolism during stress response and in cancer. Being the target of a feedback loop initiated by the pyruvate kinase substrate PEP, dynamic TPI inhibition distributes metabolites between glycolysis and the PPP [7,14]. Here we present a TPI–PEP co-crystal structure, demonstrating that PEP directly binds into the catalytic pocket of TPI. In structure–function studies involving different TPI point mutations including a rare natural variant (TPI_Ile170Val_ [22]), and two mutants designed on the basis of the crystallographic findings (TPI_Lys13Arg_ and TPI_Ile170Thr_), we have demonstrated that PEP functions as a competitive TPI inhibitor, being able to interfere with the enzymatic TPI function during catalysis. Finally, studies with transgenic yeast cells expressing these human TPI mutants revealed that low TPI activity increases PPP metabolite concentrations, increased oxidant resistance and decreased heat tolerance. Hence, the PYK–TPI feedback loop, leading to the regulation of glycolysis and the PPP to adapt to oxidative stress conditions, is the consequence of active-site competitive TPI inhibition by the PK substrate PEP.

## 5. Material and methods

Recombinant TPI expression, enzyme purification, Western blotting, yeast cultivation and strain generation were conducted according to standard procedures and are described in the electronic supplementary material. The plasmids generated in this study have been deposited at Addgene (http://www.addgene.org) and are listed in table 2.

**Table 2.** Plasmids used in this study and their deposition ID (http://www.addgene.org).

plasmid name	encoded protein	application	addgene #
p413GPD-hTPI	human wild-type TPI1	expression in *S. cerevisiae* (*HIS3*, cen)	50719
p413GPD-hTPI Ile170Val	human TPI1 Ile170Val	expression in *S. cerevisiae* (*HIS3*, cen)	50720
p413GPD-hTPI Ile170Thr	human TPI1 Ile170Thr	expression in *S. cerevisiae* (*HIS3*, cen)	50721
p413GPD-hTPI Lys13Arg	human TPI1 Lys13Arg	expression in *S. cerevisiae* (*HIS3*, cen)	50722
pET20b-hTPI	human wild-type TPI1	expression and purification in *E. coli*	50723
pET20b-hTPI Ile170Val	human TPI1 Ile170Val	expression and purification in *E. coli*	50724
pET20b-hTPI Ile170Thr	human TPI1 Ile170Thr	expression and purification in *E. coli*	50725
pET20b-hTPI Lys13Arg	human TPI1 Lys13Arg	expression and purification in *E. coli*	50726

### 5.1. Crystallization of triosephosphate isomerase–phosphoenolpyruvate complex

Native rabbit muscle TPI (TPI, Sigma) was buffer exchanged into crystallization buffer (20 mM Tris pH: 7.0, 150 mM NaCl, 5 mM MgCl_2_) with a HiTrap Desalting column and concentrated to 10 mg ml^−1^ with a VIVA spin 2 ml concentrator (MWCO: 10 kDa). PEP was added to the TPI solution to a final concentration of 5 mM. Crystals were grown at 20°C using the sitting-droplet vapour diffusion method by mixing 200 nl of TPI–PEP complex with 200 nl of reservoir solution (0.1 M MES pH: 6.5, 25% polyethylene glycol (PEG) 8000). Crystals appeared 1 day after setting up the crystallization trial and reached the final size in 1 week. The crystals were transferred briefly into reservoir solution supplemented with 25% v/v PEG 400 as cryoprotectant before flash freezing in liquid nitrogen.

### 5.2. Data collection, structure determination and refinement

X-ray diffraction data were collected at 100 K from cryoprotected crystals at beamline I24 at the Diamond Light Source. A complete dataset of TPI–PEP crystal was collected to a resolution of 1.55 Å. The data were processed and scaled using iMOSFLM and SCALA [46,47], respectively. Molecular replacement was performed with the CCP4 suite program Phaser [48] using the rabbit muscle *apo* TPI (PDB ID: 1R2R) [27] as the search model. The map identified PEP in the active site, and the initial model (without ligand) was refined using Refmac5 [49]. One protomer was manually adjusted into the electron density map using Coot and directly placed in the second protomer based on non-crystallographic symmetry. The model was refined again with TLS, NCS (non-crystallographic symmetry) and restrained refinement using Refmac5. PEP was finally built into the electron density map and then refined. A summary of the crystallographic data and refinement are given in table 1. Figures were generated using PYMOL.

### 5.3. Circular dichroism

Recordings of the far-ultraviolet (UV) CD spectrum were used to verify the native conformation of the purified TPI enzyme species. The TPI proteins were diluted to a final concentration of approximately 3.7 mM in 20 mM HEPES (pH 7.5). CD recordings were performed at 25°C on a Jobin Yvon CD6 Dichrograph, as described previously [50]. Three scans were averaged and base line subtracted using the software provided by the Jobin Yvon CD6 Dichrograph manufacturer.

### 5.4. Enzyme activity assays

TPI activity was determined as described previously [21,51]. In brief, activity of TPI in cell-free protein extracts of transgenic yeast expressing human TPI, or with purified human TPI recombinantely expressed in *E. coli*, was determined in an enzyme-coupled reaction with glycerol 3-phosphate dehydrogenase. Optical density measurements at 340 nm were used to detect NADH to NAD+ oxidation upon adding the TPI substrate glyceraldehyde 3-phosphate and recorded in 12 s intervals in a spectrophotometer (Amersham US 2000). *K*_m_ and *K*_i_ values were determined by generating saturation curves with G3P and PEP, respectively.

### 5.5. Thermal shift assays

The detection of protein thermal unfolding was performed in 96-well plates on an iQ5 real-time PCR cycler (BioRad). The reaction mix of 100 ml 20 mM HEPES (pH 7.5) contained approximately 4.5 mM protein, 0.4 ml 500× SYPRO Orange (Sigma Aldrich) and PEP and/or G3P at the indicated concentrations. Thermal unfolding of the proteins was monitored by increasing the temperature from 25 to 95°C in 2°C min^–1^ steps. Measurements were taken every 0.5°C. The resulting curves were each fitted with a four-parameter log-logistic function and protein melt points (inflection points) were calculated using R v. 2.14.1 and the drc package v. 2.3-0. The protein melt points for each ligand concentration were plotted against the melt temperature and fitted with equation (5.1).5.1
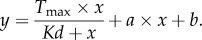


### 5.6. Oxidant-tolerance tests and growth curves

Oxidant tolerance tests were performed as described earlier [7] and growth was monitored after 2–3 days of incubation at 30°C. For growth curves, overnight cultures of the indicated yeast strains were diluted to an OD_600_ = 0.2 in SC^−His^ media. Aliquots of the same cultures were incubated for 5 min at the indicated temperatures. Immediately after the heat shock, the cells were transferred to a 96-well plate and growth was monitored in a FLUOstar OPTIMA (BMG Labtech) plate reader for 25 h.

### 5.7. Sample extraction for metabolomics

Sugar phosphate intermediates were quantified with a procedure adapted from our earlier work [38,52]. Briefly, 7.5 OD units of cell suspension were sampled in log phase at an OD_600_ 1.5 ± 0.05 by rapid cold methanol quenching [53] and then lysed in 200 µl organic extraction buffer (75 : 25 acetonitrile : water, 0.2% formic acid) by three repeated FastPrep-24 (MP Biomedicals) cycles for 20 s at 6.5 m s^−1^. The second extraction cycle was performed with 200 µl and UPLC-grade water. Supernatants from both extraction steps were combined and the solvent was evaporated in a SpeedVac concentrator. The dry pellets were resuspended in 100 µl 7% acetonitrile, centrifuged and metabolite concentrations were quantified by subsequent LC-MS/MS analysis.

### 5.8. LC-MS/MS measurements

Samples were subjected to LC separation (Agilent 1290) on a C8 column (Zorbax SB-C8 Rapid Resolution HD, 2.1 × 100 mm, 1.8 µm, Agilent; column temperature: 20°C, injection volume: 1 µl). Separation was achieved by isocratic flow at 12% acetonitrile for 3.5 min followed by a gradient to 38% acetonitrile within 2.5 min. With an additional washing step (42% acetonitrile, 0.5 min) and re-equilibration to starting conditions, this resulted in a total cycle time of 7.5 min. All buffers contained 750 mg l^−1^ octylammoniumacetate as ion pairing reagent. An online coupled triple quadrupole mass spectrometer (Agilent 6460) operating in SRM mode was used for quantification. Individual metabolites were identified by matching retention time and fragmentation pattern with commercially available standards. SRM transitions, ionization and fragmentation energies were optimized for each compound (electronic supplementary material, table S2). Ion source settings are listed in the electronic supplementary material, table S3. Data analysis was done in the Masshunter Workstation software package (Agilent). External calibration curves were measured repeatedly and used to determine absolute concentrations.

## Supplementary Material

Supplementary-Figures.pdf

## Supplementary Material

Supplementary-Material.pdf

## Data accessibility

The coordinates of the PEP-bound TPI crystallographic structure have been deposited with the PDB with code 4OWG.

## References

[1] Buescher JM 2012 Global network reorganization during dynamic adaptations of *Bacillus subtilis* metabolism. Science 335, 1099–1103. (doi:10.1126/science.1206871) 22383848 10.1126/science.1206871

[2] Grüning N-MM Lehrach H Ralser M 2010 Regulatory crosstalk of the metabolic network. Trends Biochem. Sci. 35, 220–227. (doi:10.1016/j.tibs.2009.12.001) 20060301 10.1016/j.tibs.2009.12.001

[3] Cornish-Bowden A 2013 A model of yeast glycolysis based on a consistent kinetic characterisation of all its enzymes. FEBS Lett. 587, 2832–2841. (doi:10.1016/j.febslet.2013.06.043) 23831062 10.1016/j.febslet.2013.06.043 PMC3764422

[4] Daran-Lapujade P 2007 The fluxes through glycolytic enzymes in *Saccharomyces cerevisiae* are predominantly regulated at posttranscriptional levels. Proc. Natl Acad. Sci. USA 104, 15 753–15 758. (doi:10.1073/pnas.0707476104) 17898166 10.1073/pnas.0707476104 PMC2000426

[5] Teusink B 2000 Can yeast glycolysis be understood in terms of *in vitro* kinetics of the constituent enzymes? Testing biochemistry. Eur. J. Biochem. 267, 5313–5329. (doi:10.1046/j.1432-1327.2000.01527.x) 10951190 10.1046/j.1432-1327.2000.01527.x

[6] Markus M Kuschmitz D Hess B 1985 Properties of strange attractors in yeast glycolysis. Biophys. Chem. 22, 95–105. (doi:10.1016/0301-4622(85)80030-2) 17007784 10.1016/0301-4622(85)80030-2

[7] Ralser M 2007 Dynamic rerouting of the carbohydrate flux is key to counteracting oxidative stress. J. Biol. 6, 10 (doi:10.1186/jbiol61) 18154684 10.1186/jbiol61 PMC2373902

[8] Chechik G Oh E Rando O Weissman J Regev A Koller D 2008 Activity motifs reveal principles of timing in transcriptional control of the yeast metabolic network. Nat. Biotechnol. 26, 1251–1259. (doi:10.1038/nbt.1499) 18953355 10.1038/nbt.1499 PMC2651818

[9] Godon C Lagniel G Lee J Buhler JM Kieffer S Perrot M Boucherie H Toledano MB Labarre J 1998 The H2O2 stimulon in *Saccharomyces cerevisiae*. J. Biol. Chem. 273, 22 480–22 489. (doi:10.1074/jbc.273.35.22480) 10.1074/jbc.273.35.22480 9712873

[10] Cosentino C Grieco D Costanzo V 2011 ATM activates the pentose phosphate pathway promoting anti-oxidant defence and DNA repair. Embo J. 30, 546–555. (doi:10.1038/emboj.2010.330) 21157431 10.1038/emboj.2010.330 PMC3034007

[11] Anastasiou D 2011 Inhibition of pyruvate kinase M2 by reactive oxygen species contributes to cellular antioxidant responses. Science 334, 1278–1283. (doi:10.1126/science.1211485) 22052977 10.1126/science.1211485 PMC3471535

[12] Gottlieb E Vousden KH 2010 p53 regulation of metabolic pathways. Cold Spring Harb. Perspect. Biol. 2, a001040 (doi:10.1101/cshperspect.a001040) 20452943 10.1101/cshperspect.a001040 PMC2845207

[13] Christofk HR Vander Heiden MG Harris MH Ramanathan A Gerszten RE Wei R Fleming MD Schreiber SL Cantley LC 2008 The M2 splice isoform of pyruvate kinase is important for cancer metabolism and tumour growth. Nature 452, 230–233. (doi:10.1038/nature06734) 18337823 10.1038/nature06734

[14] Gruning NM 2011 Pyruvate kinase triggers a metabolic feedback loop that controls redox metabolism in respiring cells. Cell Metab. 14, 415–427. (doi:10.1016/j.cmet.2011.06.017) 21907146 10.1016/j.cmet.2011.06.017 PMC3202625

[15] Israelsen WJ 2013 PKM2 isoform-specific deletion reveals a differential requirement for pyruvate kinase in tumor cells. Cell 155, 397–409. (doi:10.1016/j.cell.2013.09.025) 24120138 10.1016/j.cell.2013.09.025 PMC3850755

[16] Emmerling M Dauner M Ponti A Fiaux J Hochuli M Szyperski T Wuthrich K Bailey JE Sauer U 2002 Metabolic flux responses to pyruvate kinase knockout in *Escherichia coli*. J. Bacteriol. 184, 152–164. (doi:10.1128/JB.184.1.152-164.2002) 11741855 10.1128/JB.184.1.152-164.2002 PMC134756

[17] Fenton AW Reinhart GD 2009 Disentangling the web of allosteric communication in a homotetramer: heterotropic inhibition in phosphofructokinase from *Escherichia coli*. Biochemistry 48, 12 323–12 328. (doi:10.1021/bi901456p) 10.1021/bi901456p PMC2797571 19905012

[18] Lambeir A-M Opperdoes FR Wierenga RK 1987 Kinetic properties of triose-phosphate isomerase from *Trypanosoma brucei brucei*. A comparison with the rabbit muscle and yeast enzymes. Eur. J. Biochem. 168, 69–74. (doi:10.1111/j.1432-1033.1987.tb13388.x) 3311744 10.1111/j.1432-1033.1987.tb13388.x

[19] Blacklow SC Raines RT Lim WA Zamore PD Knowles JR 1988 Triosephosphate isomerase catalysis is diffusion controlled. Appendix: analysis of triose phosphate equilibria in aqueous solution by 31P NMR. Biochemistry 27, 1158–1167. (doi:10.1021/bi00404a013) 3365378 10.1021/bi00404a013

[20] Wierenga RK Kapetaniou EG Venkatesan R 2010 Triosephosphate isomerase: a highly evolved biocatalyst. Cell Mol. Life Sci. 67, 3961–3982. (doi:10.1007/s00018-010-0473-9) 20694739 10.1007/s00018-010-0473-9 PMC11115733

[21] Ralser M Heeren G Breitenbach M Lehrach H Krobitsch S 2006 Triose phosphate isomerase deficiency is caused by altered dimerization-not catalytic inactivity-of the mutant enzymes. PLoS ONE 1, e30 (doi:10.1371/journal.pone.0000030) 17183658 10.1371/journal.pone.0000030 PMC1762313

[22] Arya R Lalloz MR Bellingham AJ Layton DM 1997 Evidence for founder effect of the Glu104Asp substitution and identification of new mutations in triosephosphate isomerase deficiency. Hum. Mutat. 10, 290–294. (doi:10.1002/(SICI)1098-1004(1997)10:4<290::AID-HUMU4>3.0.CO;2-L) 9338582 10.1002/(SICI)1098-1004(1997)10:4<290::AID-HUMU4>3.0.CO;2-L

[23] Kruger A 2011 The pentose phosphate pathway is a metabolic redox sensor and regulates transcription during the antioxidant response. Antioxid. Redox Signal. 15, 311–324. (doi:10.1089/ars.2010.3797) 21348809 10.1089/ars.2010.3797

[24] Joseph-McCarthy D Lolis E Komives EA Petsko GA 1994 Crystal structure of the K12M/G15A triosephosphate isomerase double mutant and electrostatic analysis of the active site. Biochemistry 33, 2815–2823. (doi:10.1021/bi00176a010) 8130194 10.1021/bi00176a010

[25] Ralser M Nebel A Kleindorp R Krobitsch S Lehrach H Schreiber S Reinhardt R Timmermann B 2008 Sequencing and genotypic analysis of the triosephosphate isomerase (TPI1) locus in a large sample of long-lived Germans. BMC Genet. 9, 38 (doi:10.1186/1471-2156-9-38) 18510744 10.1186/1471-2156-9-38 PMC2424074

[26] Banner DW 1975 Structure of chicken muscle triose phosphate isomerase determined crystallographically at 2.5 angstrom resolution using amino acid sequence data. Nature 255, 609–614. (doi:10.1038/255609a0) 1134550 10.1038/255609a0

[27] Aparicio R Ferreira ST Polikarpov I 2003 Closed conformation of the active site loop of rabbit muscle triosephosphate isomerase in the absence of substrate: evidence of conformational heterogeneity. J. Mol. Biol. 334, 1023–1041. (doi:10.1016/j.jmb.2003.10.022) 14643664 10.1016/j.jmb.2003.10.022

[28] Jogl G Rozovsky S McDermott AE Tong L 2003 Optimal alignment for enzymatic proton transfer: structure of the Michaelis complex of triosephosphate isomerase at 1.2-Å resolution. Proc. Natl Acad. Sci. USA 100, 50–55. (doi:10.1073/pnas.0233793100) 12509510 10.1073/pnas.0233793100 PMC140880

[29] Schneider AS 2000 Triosephosphate isomerase deficiency: historical perspectives and molecular aspects. Baillieres Best Pr. Res. Clin. Haematol. 13, 119–140. (doi:10.1053/beha.2000.0061) 10.1053/beha.2000.0061 10916682

[30] Lo M-C Aulabaugh A Jin G Cowling R Bard J Malamas M Ellestad G 2004 Evaluation of fluorescence-based thermal shift assays for hit identification in drug discovery. Anal. Biochem. 332, 153–159. (doi:10.1016/j.ab.2004.04.031) 15301960 10.1016/j.ab.2004.04.031

[31] Albery WJ Knowles JR 1976 Free-energy profile of the reaction catalyzed by triosephosphate isomerase. Biochemistry 15, 5627–5631. (doi:10.1021/bi00670a031) 999838 10.1021/bi00670a031

[32] Reynolds SJ Yates DW Pogson CI 1971 Dihydroxyacetone phosphate: its structure and reactivity with glycerophosphate dehydrogenase, aldolase and triose phosphate isomerase and some possible metabolic implications. Biochem. J. 122, 285–297. 4330197 10.1042/bj1220285 PMC1176777

[33] Lodi PJ Chang LC Knowles JR Komives EA 1994 Triosephosphate isomerase requires a positively charged active site: the role of lysine-12. Biochemistry 33, 2809–2814. (doi:10.1021/bi00176a009) 8130193 10.1021/bi00176a009

[34] Picotti P Bodenmiller B Mueller LN Domon B Aebersold R 2009 Full dynamic range proteome analysis of *S. cerevisiae* by targeted proteomics. Cell 138, 795–806. (doi:10.1016/j.cell.2009.05.051) 19664813 10.1016/j.cell.2009.05.051 PMC2825542

[35] Yamaji R Fujita K Nakanishi I Nagao K Naito M Tsuruo T Inui H Nakano Y 2004 Hypoxic up-regulation of triosephosphate isomerase expression in mouse brain capillary endothelial cells. Arch. Biochem. Biophys. 423, 332–342. (doi:10.1016/j.abb.2004.01.003) 15001397 10.1016/j.abb.2004.01.003

[36] Morgan HP O'Reilly FJ Wear MA O'Neill JR Fothergill-Gilmore LA Hupp T Walkinshaw MD 2013 M2 pyruvate kinase provides a mechanism for nutrient sensing and regulation of cell proliferation. Proc. Natl Acad. Sci. USA 110, 5881–5886. (doi:10.1073/pnas.1217157110) 23530218 10.1073/pnas.1217157110 PMC3625322

[37] Bluemlein K Gruning NM Feichtinger RG Lehrach H Kofler B Ralser M Grüning N-M 2011 No evidence for a shift in pyruvate kinase PKM1 to PKM2 expression during tumorigenesis. Oncotarget 2, 393–400. 21789790 10.18632/oncotarget.278 PMC3248187

[38] Wamelink M Jansen E Struys E Lehrach H Jakobs C Ralser M 2009 Quantification of *Saccharomyces cerevisiae* pentose-phosphate pathway intermediates by LC-MS/MS. Nat. Protoc. Netw. See http://www.nature.com/protocolexchange/protocols/560. (doi:10.1038/nprot.2009.140)

[39] Wamelink MM Smith DE Jakobs C Verhoeven NM 2005 Analysis of polyols in urine by liquid chromatography-tandem mass spectrometry: a useful tool for recognition of inborn errors affecting polyol metabolism. J. Inherit. Metab. Dis. 28, 951–963. (doi:10.1007/s10545-005-0233-4) 16435188 10.1007/s10545-005-0233-4

[40] Mulleder M Capuano F Pir P Christen S Sauer U Oliver SG Ralser M 2012 A prototrophic deletion mutant collection for yeast metabolomics and systems biology. Nat. Biotechnol. 30, 1176–1178. (doi:10.1038/nbt.2442) 23222782 10.1038/nbt.2442 PMC3520112

[41] Shenton D Grant CM 2003 Protein S-thiolation targets glycolysis and protein synthesis in response to oxidative stress in the yeast *Saccharomyces cerevisiae*. Biochem. J. 374, 513–519. (doi:10.1042/BJ20030414) 12755685 10.1042/BJ20030414 PMC1223596

[42] Nogae I Johnston M 1990 Isolation and characterization of the ZWF1 gene of *Saccharomyces cerevisiae*, encoding glucose-6-phosphate dehydrogenase. Gene 96, 161–169. (doi:10.1016/0378-1119(90)90248-P) 2269430 10.1016/0378-1119(90)90248-p

[43] Stanford DR Whitney ML Hurto RL Eisaman DM Shen WC Hopper AK 2004 Division of labor among the yeast Sol proteins implicated in tRNA nuclear export and carbohydrate metabolism. Genetics 168, 117–127. (doi:10.1534/genetics.104.030452) 15454531 10.1534/genetics.104.030452 PMC1448090

[44] Benjaphokee S Koedrith P Auesukaree C Asvarak T Sugiyama M Kaneko Y Boonchird C Harashima S 2012 CDC19 encoding pyruvate kinase is important for high-temperature tolerance in *Saccharomyces cerevisiae*. New Biotechnol. 29, 166–176. (doi:10.1016/j.nbt.2011.03.007) 10.1016/j.nbt.2011.03.007 21459167

[45] Kahm M Hasenbrink G Lichtenberg-Fraté H Ludwig J Kschischo M 2010 grofit: fitting biological growth curves with R. *J. Stat. Softw.* **33**. See http://www.jstatsoft.org/v33/i07.

[46] Battye TGG Kontogiannis L Johnson O Powell HR Leslie AGW 2011 iMOSFLM: a new graphical interface for diffraction-image processing with MOSFLM. Acta Crystallogr. D. Biol. Crystallogr. 67, 271–281. (doi:10.1107/S0907444910048675) 21460445 10.1107/S0907444910048675 PMC3069742

[47] Winn MD 2011 Overview of the CCP4 suite and current developments. Acta Crystallogr. D. Biol. Crystallogr. 67, 235–242. (doi:10.1107/S0907444910045749) 21460441 10.1107/S0907444910045749 PMC3069738

[48] McCoy AJ Grosse-Kunstleve RW Adams PD Winn MD Storoni LC Read RJ 2007 Phaser crystallographic software. J. Appl. Crystallogr. 40, 658–674. (doi:10.1107/S0021889807021206) 19461840 10.1107/S0021889807021206 PMC2483472

[49] Murshudov GN Skubák P Lebedev AA Pannu NS Steiner RA Nicholls RA Winn MD Long F Vagin AA 2011 REFMAC5 for the refinement of macromolecular crystal structures. Acta Crystallogr. D. Biol. Crystallogr. 67, 355–367. (doi:10.1107/S0907444911001314) 21460454 10.1107/S0907444911001314 PMC3069751

[50] Assenberg R Webb M Connolly E Stott K Watson M Hobbs J Thomas JO 2008 A critical role in structure-specific DNA binding for the acetylatable lysine residues in HMGB1. Biochem. J. 411, 553–561. (doi:10.1042/BJ20071613) 18241198 10.1042/BJ20071613

[51] Maitra PK Lobo Z 1971 A kinetic study of glycolytic enzyme synthesis in yeast. J. Biol. Chem. 246, 475–488. 5542016

[52] Wamelink MMC Struys EA Huck JHJ Roos B van der Knaap MS Jakobs C Verhoeven NM 2005 Quantification of sugar phosphate intermediates of the pentose phosphate pathway by LC-MS/MS: application to two new inherited defects of metabolism. J. Chromatogr. B Anal. Technol. Biomed. Life Sci. 823, 18–25. (doi:10.1016/j.jchromb.2005.01.001) 10.1016/j.jchromb.2005.01.001 16055050

[53] De Koning W van Dam K 1992 A method for the determination of changes of glycolytic metabolites in yeast on a subsecond time scale using extraction at neutral pH. Anal. Biochem. 204, 118–123. (doi:10.1016/0003-2697(92)90149-2) 1514678 10.1016/0003-2697(92)90149-2

