# Supplementary material for: Inhibition of triosephosphate isomerase by phosphoenolpyruvate in the feedback-regulation of glycolysis
Source: Open Biol. 2014 Mar 5;4(3):130232. doi: 10.1098/rsob.130232 (PMC3971408; doi:10.1098/rsob.130232)
Supplement: Supplementary-Figures.pdf [file rsob130232supp1.docx]

Grüning et al.
**The structural basis for inhibition of triosephosphate isomerase by phosphoenolpyruvate in the feedback regulation of glycolysis**

**Supplemental Information (SI)**

**Supplemental Methods**

***Cloning, Recombinant TPI expression and purification***

DNA fragments encoding human TPI were obtained by PCR using the p413GPD-TPI and p413GPD-TPI_Ile170Val_ plasmids (1) as templates, human TPI _Ile170Thr_ and TPI _Lys13Arg_ alleles were generated by site directed PCR mutagenesis. The DNA fragments were ligated into the pET20b expression vector to generate N-terminal 6x His-tagged proteins, and into p413GPD for yeast expression plasmids (2). All plasmids were verified by sequencing and primer sequences are given in Suppl. Table 1. The plasmids used in this study have been deposited ad Addgene (Table 2, http://www.addgene.org).

For protein production in *E. coli*, BL21(D3) cells were transformed with the pET20b based plasmids, and cultured in 2xYT medium containing Carbenicillin (100 μg ml^-1^). Protein expression was induced at A_600_=0.5 by adding 0.5 mM isopropyl beta-d-1-thiogalactopyranoside (IPTG). The 3 litres cell cultures were further incubated at 25°C for 15 hours. Cells were harvested by centrifugation and resuspended in lysis buffer (20 mM Tris pH 8.0, 150 mM NaCl, 5 mM MgCl_2_) supplemented with complete EDTA-free protease inhibitor and 5 U ml^-1^ DNaseI. Cell disruption was carried out by 5 passages through a high-pressure homogenizer (Emulsiflex). The lysate was cleared from cell debris by centrifugation at 31 500 rpm for 2 hours at 4°C. For pre-purification by ion exchange chromatography, the cleared cell lysate was supplemented with sodium chloride to a final concentration of 300 mM and loaded on a 5 ml HisTrap HP column, washed with lysis buffer supplemented with 30 mM imidazole, then eluted in lysis buffer supplemented with 500 mM imidazole. The fractions collected from the ion exchange column were pooled and concentrated by centrifugation to 2 ml using a VIVA Spin filter (MWCO=30 kDa). For final purification, the concentrated protein solution was loaded on a HiLoad 16/60 Superdex 75^TM^ prep grade column and eluted with lysis buffer at a flow rate of 1 ml min^-1^. The purity of the collected TPI solution was verified by Coomassie staining of SDS gels.

***Yeast cultivation and strain generation***

Yeast were grown at 28-30°C either in yeast-extract peptone 2% glucose (YPD) or in synthetic complete (SC) media lacking the indicated amino acids/bases as described recently (3). The yeast parent strain which is endogenously deleted for *TPI1* (*YDR050CΔ0::LEU2),* the centromeric yeast expression vectors encoding for human TPI and TPI _Ile170Val_ (1) as well as yeast strains *Δzwf1Δtpi1, Δsol3Δtpi1,* and *Δsol4Δtpi1* (4) were described earlier. Isogenic TPI mutants were generated by plasmid shuffling. *Δtpi1* yeast was transformed with an URA3-plasmid encoding for wild-type TPI (p416GPD-TPI ). The *Δtpi1* p416GPD-TPI strain was subsequently transformed with *HIS3* marked plasmids carrying the mutant alleles (p413GPD-TPI, p413GPD-TPI _Ile170Val_, p413GPD-TPI _Ile170Thr_, p413GPD-TPI _Lys13Arg_). Finally, the URA3-plasmid was counter-selected for positive transformants on SC^-His^ containing 0.15% 5’FOA. Obtained transformants were validated for the loss of the *URA3* plasmid and transformed with the pHLUM minichromosome (3) to restore prototrophy.

***Western Blotting***

Yeast cultures were harvested by centrifugation at mid-exponential growth. The cell pellets were disrupted with glass beads in PBS on a FastPrep Device (MP) and then cleared from cell debris by centrifugation. For each cell extract the total protein concentration was determined using a Bradford assay (Biorad) and adjusted in PBS to the same protein concentration for all samples analysed. Western blotting and Ponceau Red staining was then conducted following standard procedures, using a PVDF membrane (GE Healthcare Amersham Hybond^TM^-P). TPI antiserum was generated as described previously (5).

**Supplementary Table S1 Primer sequences used for mutagenesis and cloning**

| **Primer Name** | **Primer Sequence** | | **Reference, usage** |
| --- | --- | --- | --- |
| TPI Ile170Thr fw | gcctgtgtgggcca**c**tggtactg | | this study, mutagenesis |
| TPI Ile170Thr rev | cagtacca**g**tggcccacacaggc | | this study, mutagenesis |
| TPI Lys14Arg fw | ggaaactgga**g**gatgaacgg | | this study, mutagenesis |
| TPI Lys14Arg rev | ccgttcat**c**ctccagtttcc | | this study, mutagenesis |
| TPI-CDS-fw-BamH1 | gaggatccatggcgccctccaggaagtt | | (1), cloning |
| TPI-CDS-rev-Xho1 | tcgactcgagtcattgtttggcattgatga | | (1), cloning |
| exchanged bases are indicated in bold, underlined DNA sequences indicate introduced restriction sites | |  | |

**Supplementary Table 2 SRM transitions and mass spectrometer parameters**

| **Name** | **Sum formula** | **Exact Mass**  **g/mol** | **Transition** | **Frag-**  **mentor** | **Collision energy** | **ESI Mode** |
| --- | --- | --- | --- | --- | --- | --- |
| **Glucose** | C_6_H_12_O_6_ | 180.0634 | 179.0 -> 89.0 | 70 | 1 | Negative |
| **Pyr** | C_3_H_4_O_3_ | 88.01604 | 87.0 -> 43.0 | 55 | 3 | Negative |
| **S7P** | C_7_H_15_O_10_P | 290.0402 | 289.0 -> 97.0 | 100 | 12 | Negative |
| **G6P** | C_6_H_13_O_9_P | 260.0297 | 259.0 -> 97.0 | 100 | 12 | Negative |
| **X5P/Ru5P** | C_5_H_9_O_8_P | 230.0191 | 229.0 -> 97.0 | 85 | 12 | Negative |
| **F6P** | C_6_H_13_O_9_P | 260.0297 | 259.0 -> 97.0 | 100 | 12 | Negative |
| **E4P** | C_4_H_9_O_7_P | 200.0085 | 199.0 -> 97.0 | 70 | 6 | Negative |
| **G3P** | C_3_H_7_O_6_P | 169.9980 | 169.0 -> 97.0 | 70 | 5 | Negative |
| **R5P** | C_5_H_9_O_8_P | 230.0192 | 229.0 -> 97.0 | 85 | 12 | Negative |
| **DHAP** | C_3_H_7_O_6_P | 169.9980 | 169.0 -> 97.0 | 70 | 5 | Negative |
| **6PG** | C_6_H_13_O_10_P | 276.0246 | 275.0 -> 97.0 | 100 | 18 | Negative |
| **2-PG/3-PG** | C_3_H_7_O_7_P | 185.9929 | 185.0 -> 97.0 | 75 | 11 | Negative |
| **PEP** | C_3_H_5_O_6_P | 167.9824 | 167.0 -> 79.0 | 50 | 7 | Negative |
| **F16BP** | C_6_H_14_O_12_P_2_ | 339.9960 | 339.0 -> 97.0 | 175 | 16 | Negative |

Abbreviations: G6P: glucose 6-phosphate; F6P: fructose 6-phosphate; F1,6BP: fructose 1,6-bisphosphate; DHAP dihydroxyacetone phosphate, G3P glyceraldehyde 3-phosphate; 3PG: 3-phosphoglycerate; 2PG: 2-phosphoglycerate; PEP: phosphoenolpyruvate; Pyr: Pyruvate. *PPP*: 6PG: 6-phosphogluconate; RI5P: Ribulose 5-phosphate; R5P: Ribose 5-phosphate; X5P: Xylulose 5-phosphate, S7P: sedoheptulose 7-phosphate, E4P: erythrose 4-phosphate. Ru5P and X5P, 2-PG and 3-PG and non-phosphorylated hexose sugars co-eluted with the same RT and were quantified in pools.

**Supplementary Table 3: Ion source settings, Agilent 6460**

| **Name** | **Value** |
| --- | --- |
| **Scan Type** | MRM (SRM) |
| **Cell Acceleration voltage** | 7 V |
| **Gas flow** | 8 l min^-1^ |
| **Gas temperature** | 300°C |
| **Sheath gas flow** | 11 l min^-1^ |
| **Sheath gas temperature** | 300°C |
| **Nebulizer** | 50 psi (nitrogen) |
| **Negative**  **Capillary voltage** | 3000 V |
| **Nozzle voltage** | 500 V |

**Supplementary References**

1. Ralser M, Heeren G, Breitenbach M, Lehrach H, Krobitsch S (2006) Triose Phosphate Isomerase Deficiency Is Caused by Altered Dimerization-Not Catalytic Inactivity-of the Mutant Enzymes. *PLoS One* 1:e30.

2. Mumberg D, Muller R, Funk M (1995) Yeast vectors for the controlled expression of heterologous proteins in different genetic backgrounds. *Gene* 156:119–122.

3. Mulleder M et al. (2012) A prototrophic deletion mutant collection for yeast metabolomics and systems biology. *Nat Biotechnol* 30:1176–1178.

4. Ralser M et al. (2007) Dynamic rerouting of the carbohydrate flux is key to counteracting oxidative stress. *J Biol* 6:10.

5. Yamaji R et al. (2004) Hypoxic up-regulation of triosephosphate isomerase expression in mouse brain capillary endothelial cells. *Arch Biochem Biophys* 423:332–342.
