# Supplementary material for: Inhibition of triosephosphate isomerase by phosphoenolpyruvate in the feedback-regulation of glycolysis
Source: Open Biol. 2014 Mar 5;4(3):130232. doi: 10.1098/rsob.130232 (PMC3971408; doi:10.1098/rsob.130232)
Supplement: Supplementary-Material.pdf [file rsob130232supp2.pdf]

Nana-Maria Grüning, Dijun Du, Markus A Keller , Ben F Luisi and Markus Ralser

The inhibition of triosephosphate isomerase by phosphoenolpyruvate in the feedback-regulation of glycolysis

Supplementary Figures

S1

catalytically active sites   mutated sites   difference human-rabbit

```
human    APSRKFFVGGNWKMNGRKQSLGELIGTLNAAKVPADTEVVCAPPTAYIDF 50
rabbit   APSRKFFVGGNWKMNGRKKNLGELITTLNAAKVPADTEVVCAPPTAYIDF 50
         ******************:.***** ************************

human    ARQKLDPKIAVAAQNCYKVTNGAFTGEISPGMIKDCGATWVVLGHSERRH 100
rabbit   ARQKLDPKIAVAAQNCYKVTNGAFTGEISPGMIKDCGATWVVLGHSERRH 100
         **************************************************

human    VFGESDELIGQKVAHALAEGLGVIACIGEKLDEREAGITEKVVFEQTKVI 150
rabbit   VFGESDELIGQKVAHALSEGLGVIACIGEKLDEREAGITEKVVFEQTKVI 150
         *****************:********************************

human    ADNVKDWSKVVLAYEPVWAIGTGKTATPQQAQEVHEKLRGWLKSNVSDAV 200
rabbit   ADNVKDWSKVVLAYEPVWAIGTGKTATPQQAQEVHEKLRGWLKSNVSDAV 200
         **************************************************

human    AQSTRIIYGGSVTGATCKELASQPDVDGFLVGGASLKPEFVDIINAKQ 248
rabbit   AQSTRIIYGGSVTGATCKELASQPDVDGFLVGGASLKPEFVDIINAKQ 248
         ************************************************
```

Fig S1: Alignment of human and rabbit TPI, as well as overview of catalytic and mutant residues

The alignment of human TPI (Swiss-Prot: P60174) and rabbit TPI (Swiss-Prot: P00939) amino acid sequences was generated with an EMBL-EBI clustalW. The catalytically active residues are highlighted in green and the residues that were experimentally exchanged are indicated in purple. Human and Rabbit TPI are virtually identical, and differ in four non-catalytic residues.

S2

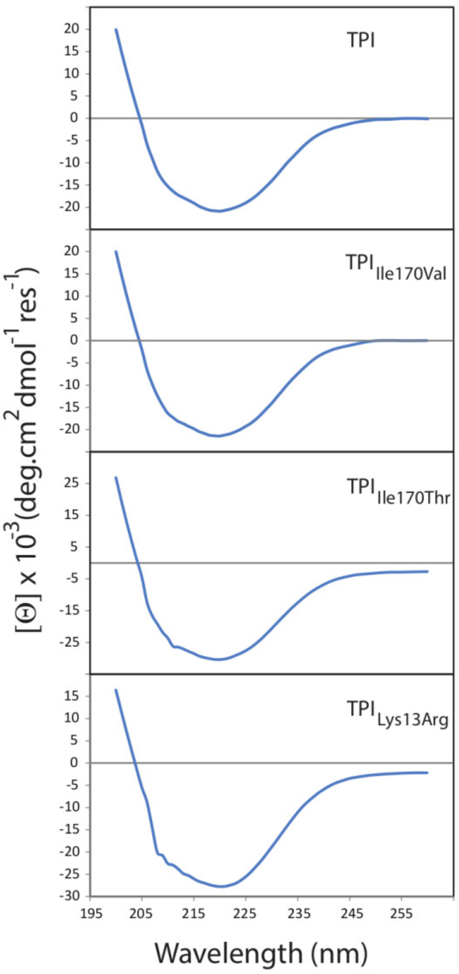

Fig S2: Far UV-Circular Dichroism spectra for purified TPI, $TPI_{Ile170Val}$, $TPI_{Ile170Thr}$ and $TPI_{Lys13Arg}$

The secondary structures of the four purified proteins were measured in 20 mM HEPES buffer, pH 7.5. The CD spectra indicate similar secondary structure composition of TPI enzymes, indicating correct folding of the TPI mutants.

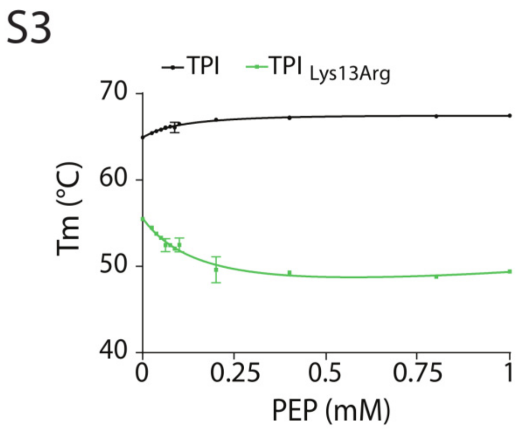

Fig S3: TPI $_{Lys13Arg}$ is thermally unstable.

Thermal melt assays upon adding increased concentrations of PEP to wild-type TPI and TPI $_{Lys13Arg}$. TPI $_{Lys13Arg}$ was thermally unstable, and PEP further destabilized the mutant enzyme.

S4

	G6P		F6P		F1,6BP		DHAP	
	AVR	STD	AVR	STD	AVR	STD	AVR	STD
TPI	106.75	5.30	75.38	25.55	223.63	21.97	33.57	29.41
TPI $_{Ile170Val}$	84.64	6.42	40.90	2.83	260.59	25.80	107.15	31.22
TPI $_{Ile170Thr}$	78.05	5.81	72.58	20.67	252.67	16.49	76.15	34.93

	G3P		2,3-PG		PEP		Pyr	
	AVR	STD	AVR	STD	AVR	STD	AVR	STD
TPI	7.15	1.27	20.03	1.85	2.13	0.30	90.93	15.01
TPI $_{Ile170Val}$	4.31	0.76	17.38	4.61	1.60	0.29	83.40	5.58
TPI $_{Ile170Thr}$	7.74	0.66	19.41	2.41	1.44	0.40	87.80	12.52

	6PG		R5P		Rl5P + X5P		S7P	
	AVR	STD	AVR	STD	AVR	STD	AVR	STD
TPI	5.82	0.80	1.31	0.25	3.23	1.08	76.98	10.45
TPI $_{Ile170Val}$	7.30	2.89	1.95	0.31	4.96	1.34	82.48	20.36
TPI $_{Ile170Thr}$	9.94	1.27	1.64	0.59	5.14	1.69	100.73	13.13

G6P, glucose 6-phosphate, **F6P,** fructose 6-phosphate, **F1,6BP,** fructose 1,6-phosphate, **DHAP,** Dihydroxyacetone phosphate, **G3P,** glyceraldehyde 3-phosphate, 2,3-PG, 2-phosphoglycerate + 3-phosphoglycerate, **PEP,** phosphoenolpyruvate, **Pyr,** pyruvate, **6PG,** 6-phosphogluconate, **R5P,** ribose 5-phosphate, **Rl5P + X5P,** ribulose 5-phosphate + xylulose 5-phosphate, **S7P,** seduheptulose 7-phosphate, **AVR,** average, **STD,** standard deviation. Concentrations are given in μM in a 100 μl cell extract.

Fig S4: Absolute concentrations

Glycolytic and PPP intermediate concentrations as determined by LC-SRM in yeast extracts expressing human wild-type TPI, TPI $_{Ile170Val}$ or TPI $_{Ile170Thr}$ in micromol / ml*OD_{600}; n=3
